# Extrinsic LiDAR/Ground Calibration Method Using 3D Geometrical Plane-Based Estimation

**DOI:** 10.3390/s20102841

**Published:** 2020-05-16

**Authors:** Mohammad Ali Zaiter, Régis Lherbier, Ghaleb Faour, Oussama Bazzi, Jean-Charles Noyer

**Affiliations:** 1 Laboratoire d’Informatique Signal et Image de la Côte d’Opale, Université du Littoral Côte d’Opale, LISIC, 59183 Dunkerque, France; mohammad-ali.zaiter@etu.univ-littoral.fr (M.A.Z.); regis.lherbier@univ-littoral.fr (R.L.); 2 Remote Sensing Research Center, National Council of Scientific Research (CNRS-L), Mansouriyeh 22411, Lebanon; gfaour@cnrs.edu.lb; 3 Department of Physics and Electronics, Faculty of Science, Lebanese University, Hadath 11-8281, Lebanon; obazzi@ul.edu.lb

**Keywords:** 3D LiDAR, laser rangefinder, extrinsic calibration, road surface object detection

## Abstract

This paper details a new extrinsic calibration method for scanning laser rangefinder that is precisely focused on the geometrical ground plane-based estimation. This method is also efficient in the challenging experimental configuration of a high angle of inclination of the LiDAR. In this configuration, the calibration of the LiDAR sensor is a key problem that can be be found in various domains and in particular to guarantee the efficiency of ground surface object detection. The proposed extrinsic calibration method can be summarized by the following procedure steps: fitting ground plane, extrinsic parameters estimation (3D orientation angles and altitude), and extrinsic parameters optimization. Finally, the results are presented in terms of precision and robustness against the variation of LiDAR’s orientation and range accuracy, respectively, showing the stability and the accuracy of the proposed extrinsic calibration method, which was validated through numerical simulation and real data to prove the method performance.

## 1. Introduction

### 1.1. Overview

With the evolution of technology, the 3D intelligent sensors have posed great challenges in signal processing, especially in their outstanding acquisition performance even in rough environment. Moving to road networks maintenance and transportation safety, the responsibility imposes itself in detecting and locating the road distortion (cracking, patching, potholes, rutting, shoving, etc.). The literature review in [1] presents different automated detection experiments and extensive research conducted on pavement adversity in recent years. The work shows the importance and the incredible progress of 3D sensors compared with the other sensors, especially the laser profiler that is characterized by its high precision measurement capability, high spatial resolution and acquisition flexibility.

In a related context, road defects present a big danger on the traffic circulation ending up with possible traffic accidents. Some traffic accidents result from the presence of disabilities or small obstacles on the roads. In fact, this is one of the major problems that people suffer from in their daily lives. The key problem in this research concerns the characterization of the road surface by detection, localization, and tracking the presence of potentially dangerous areas and road defects using a 3D LiDAR sensor. Although 2D LiDAR sensors can provide 3D data, they require the use of an additional instrument in the form of a tilt unit.

Various promising applications, which rely on LiDAR sensors, are developed in different fields: intelligent transportation systems, mobile robotics, and connected vehicles. Thus, LiDAR is a fundamental sensor contributing in multi-vehicles tracking [2], simultaneous localization and mapping [3,4], road and road boundaries detection [5,6], autonomous vehicles [7,8], recognition [9,10], and 3D reconstruction [11,12]. Almost all of these applications appear in world challenges such as DARPA Urban and Grand Challenge [13,14,15,16,17].

Basically, LiDAR sensor operation relies on the calibration process which improves the defect detection and other study procedure. Two different types of calibration exist: intrinsic and extrinsic calibration. The intrinsic calibration considers the modeling between the beam creation and measurement of the environment to estimate the sensor–environment relationship in terms of the sensor internal parameters. On the other hand, the extrinsic calibration considers the determination of the relationship between the sensor frame and the world reference frame by rotation and translation transformation.

### 1.2. Related Works

Numerous authors investigated intrinsic and extrinsic calibration methods in LiDAR sensors. An intrinsic calibration technique is presented in [18]. The calibration process is based on an optimization method, where the calibration pattern is a wide planar wall on a flat surface scanned using Velodyne HDL-64E. In addition, Glennie and Lichti [19] presented a static calibration technique to derive an optimal solution for the laser’s intrinsic calibration parameters by a planar feature-based least squares in advantage of minimal constrained network. The study in [20] shows a correlation between the internal operating temperature of the LiDAR and the Laser scanner ranging error (intrinsic parameter). The calibration process considers a planar calibration approach to estimate the internal parameters for Velodyne VLP-16.

On the other hand, an extrinsic calibration technique is presented in [21]. In this technique, a flat plane is used for the calibration and an algorithm based on the inequality of two symmetric rays in azimuth with respect to the origin is proposed. This inequality is due to the shift angle of the center line. Another extrinsic calibration technique is presented in [22], where the authors worked on a 2D laser scanner and on the rotating platform to extract the rotation axis and radius using point-plane constraint. The Levenberg–Marquardt optimization method is applied in the two above extrinsic calibration methods to solve the non-linear least squares function problem.

In [23], a numerical algorithm is presented to compute both of the intrinsic and extrinsic parameters by minimizing the systematic errors due to the geometric calibration factors. Another approach introduced in [24] computes the intrinsic and extrinsic parameters of LiDAR sensor (Velodyne HDL-64E) by unsupervised calibration for each of multi-laser beams. An optimization function seeking to minimize the point-to-plane iterated closest point is then proposed.

### 1.3. Proposed Method

In transport applications, many articles use LiDAR to detect and track objects of interest (vehicle, pedestrians, etc.) from 3D measurements. The LiDAR sensor is also used to detect the road, often in addition to camera sensors. In these applications, the idea is to have a thorough view of the driver’s environment over the widest possible horizon. This therefore involves a LiDAR sensor with a low angle of inclination (horizontally oriented sensor).

The perspective of our paper is to propose a calibration method (and road plane estimation) that works under difficult experimental conditions (high angle of inclination). Indeed, we aim at developing a calibration method that allows to determine precisely the road plane in a very close vicinity of the vehicle. The idea in the long term is to detect road defects when driving on the road network. Although developed with this in mind (i.e., with a high degree of accuracy in determining the road plan), our method is general enough to be applicable in any wider operational context.

In the context of this study (road defects detection), the LiDAR sensor is rotated toward the ground to increase the points’ density covering the defects by the multi elevation laser. This causes a complicated modification in the ground 3D view scene with respect to the LiDAR frame. Therefore, extrinsic calibration was adopted in order to transform the LiDAR frame into a global reference frame, thus modifying the ground impact points transformation into an understandable view scene.

To attain the above key objective, a first method was applied by Zaiter et al. [25] on simulation data, which was restricted to the Euler angles estimation. In this conference paper, we proposed a first approach to extrinsic calibration of LRF sensors. It has been developed for the Velodyne VLP-16 LiDAR and the theoretical approach have been evaluated on some simulation results.

This paper addresses a new flexible extrinsic calibration method as compared to the previous plane-based methods. The approach developed is generalized to all types of scanning laser rangefinders. It now presents an optimized estimation of all extrinsic calibration parameters (angles and height). This global method can be implemented on different LiDAR sensors (low-cost 3D and full 3D) with various range accuracy. In addition, the proposed technique out performs in high orientation scenarios, which is a very interesting and challenging task that aims to increase the points’ density coverage. The proposed calibration method can be summarized by the following two-fold contributions: (1) ground plane model estimation; and (2) rotation transformation matrix estimation from world ground reference to LiDAR sensor frame. The 3D Euler’s angles (sensor orientation) and the height (sensor altitude above the ground) are two essential extrinsic parameters required to calibrate the full 3D LiDAR sensors, in order to improve the capability of road defect detection as explained in Section 2.1. In addition, the problem is modeled by 4-DOF (degree of freedom) transformation, namely 3-DOF rotation and 1-DOF height, instead of 6-DOF transformation, namely 3-DOF rotation and 3-DOF translation. This modeling advantage provides the simplicity in the optimization process of the extrinsic parameters.

The structure of this paper is as follows. Section 2 presents the correlation between extrinsic parameters and the geometrical pattern reflection on the ground, the rotated multi-laser beams projection modeling on the ground and the associated measurement errors on the 3D points cloud position. Section 3 presents in detail the different steps of the proposed LiDAR/Ground Calibration Method. Then, the method is evaluated on LiDAR’s synthetic and real data in Section 4.

## 2. LiDAR/Ground Geometrical Impact Modeling

The synthetic data are generated depending on the features of multi-laser rangefinder or 3D LiDAR sensor, where the environment impact points can be modeled as an intersection between the LiDAR laser beams and the environmental surrounding surfaces. In this work, the LiDAR sensor must be oriented toward the ground to study the road defects. Therefore, the LiDAR laser beams are represented as straight lines and the ground surface as a flat plane in a 3D frame.

**Figure 1:**
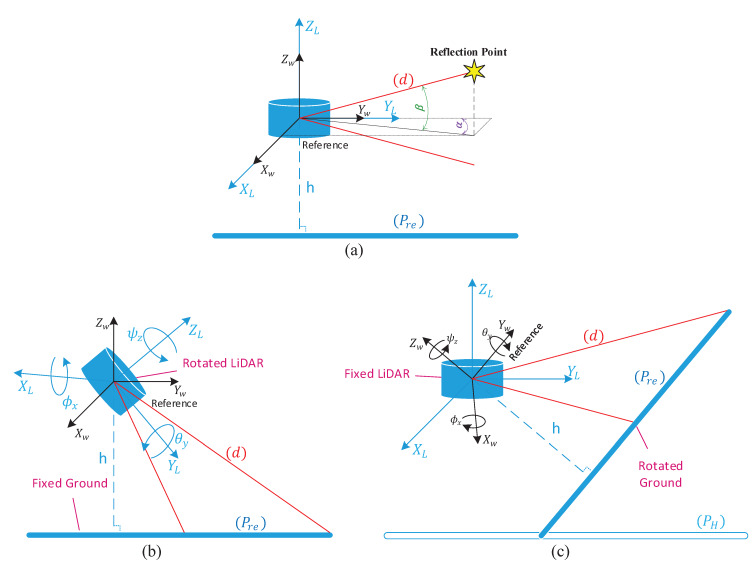
(**a**) No orientation; (**b**) practical orientation concept; and (**c**) scientific orientation concept.

Depending on the application situation, two concepts can represent the geometrical reflection model between the LiDAR sensor and the ground surface, as shown in Figure 1:

- Practical orientation concept: The LiDAR laser beams (d) are supposed to be rotated and the ground’s real plane $\left(P_{re}\right)$ is a fixed horizontal plane, as shown in Figure 1b.
- Scientific orientation concept: The LiDAR laser beams (d) are supposed to be fixed and the virtual horizontal ground surface $\left(P_{H}\right)$ must be rotated by the LiDAR’s inverse orientation in the practical concept, to get the real oblique ground plane $\left(P_{re}\right)$ in LiDAR frame, as shown in Figure 1c.

### 2.1. Extrinsic Parameters vs. Practical Concept

**Figure 2:**
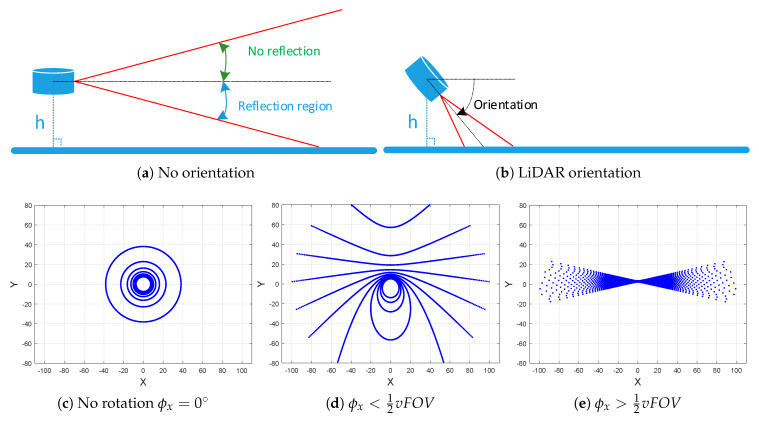
Large sparsity area vs. high density points.

The four extrinsic parameters (altitude and orientation angles) affect the research goals, where the orientation parameter is the strongest influence factor on the ground points distribution process, as shown in Figure 2a,b. The proposed calibration method must satisfy two contradictory conditions in relation to the final research objectives:

- Goal: The plane-based extrinsic calibration needs large sparsity area to improve the plane estimation, which requires high altitude and low orientation angles.
- Constraint: The stated finality of road surface object detection needs high density points to improve the capability of defect coverage points, which requires low altitude and high orientation angles.

Therefore, a trade-off is needed to optimize the extrinsic parameters (altitude and orientation angles), providing the suitable coverage points distribution over the ground.

Four geometric view patterns are summarized in three cases depending on the variation of pitch angle $\phi_{x}$ with respect to the LiDAR’s vertical field of view “vFOV”, as shown in Figure 2: (1) circles patterns (Figure 2c); (2) combination of ellipses, parabola, and hyperbolas patterns (Figure 2d); and (3) hyperbolas patterns (Figure 2e).

The study case in this problem focuses on the hyperbolas case, as shown in Figure 2e, in order to increase the points density on the ground.

### 2.2. LiDAR Laser Beams and Oblique Ground Surface Intersection

In the following part, the scientific orientation concept is chosen to model the reflections of the laser beams (d) on the real oblique ground surface $\left(P_{re}\right)$. Therefore, the LiDAR is supposed to be fixed and the parametric equations of the fixed straight lines (d) in LiDAR frame are given by:

(1) $$\left(d\right):\left\{\begin{array}{c} \left[\begin{array}{c} x\\ y\\ z \end{array}\right]=\left[\begin{array}{c} t*\tan\alpha \\ t\\ t*\sqrt{\tan^{2}\alpha +1}*\tan\beta \end{array}\right]\;\mathrm{for}\;\left\{\begin{array}{c} 0^{\circ }<\alpha <90^{\circ }\;or\\ 270^{\circ }<\alpha <360^{\circ }\\ -90^{\circ }<\beta <90^{\circ }\\ t\geq 0 \end{array}\right.\\ \left[\begin{array}{c} x\\ y\\ z \end{array}\right]=\left[\begin{array}{c} t*\tan\alpha \\ t\\ -t*\sqrt{\tan^{2}\alpha +1}*\tan\beta \end{array}\right]\;\mathrm{for}\;\left\{\begin{array}{c} 90^{\circ }<\alpha <270^{\circ }\\ -90^{\circ }<\beta <90^{\circ }\\ t\leq 0 \end{array}\right. \end{array}\right.$$

where α and β describe the azimuth and the elevation angles of each laser beam and *t* is the parameter of the parametric representation.

The virtual horizontal ground plane $\left(P_{H}\right)$ must be rotated by the 3D Euler’s angles $\psi_{z},\theta_{y},\phi_{x}$, so that the equation of the rotated real ground plane $\left(P_{re}\right)$ is expressed as a function of the horizontal ground plane $\left(P_{H}\right)$ with height *h* and rotational matrix $R_{z,y,x}\left(\psi_{z},\theta_{y},\phi_{x}\right)$. This transformation is expressed as:

(2) $$P_{re}=R_{z,y,x}\left(\psi_{z},\theta_{y},\phi_{x}\right)P_{H}$$

The parametric and Cartesian equations of the horizontal ground plane $\left(P_{H}\right)$ are expressed as follows:

(3) $$\left(P_{H}\right):\left\{\begin{array}{cc} \left[\begin{array}{c} x\\ y\\ z \end{array}\right]=\left[\begin{array}{c} t+aw\\ t+bw\\ -h \end{array}\right]\;\mathrm{parametric}\;\mathrm{equation} & \forall a,b\in R\\ z+h=0\;\mathrm{Cartesian}\;\mathrm{equation} \end{array}\right.$$

The rotational matrix $R_{z,y,x}\left(\psi_{z},\theta_{y},\phi_{x}\right)$ is expressed as:

(4) $$\begin{array}{c} R_{z,y,x}\left(\psi_{z},\theta_{y},\phi_{x}\right)=R_{z}\left(\psi_{z}\right)R_{y}\left(\theta_{y}\right)R_{x}\left(\phi_{x}\right)\\ =\left[\begin{array}{ccc} \cos\psi_{z}\cos\theta_{y} & \begin{array}{c} \cos\psi_{z}\sin\theta_{y}\sin\phi_{x}\\ -\sin\psi_{z}\cos\phi_{x} \end{array} & \begin{array}{c} \cos\psi_{z}\sin\theta_{y}\cos\phi_{x}\\ +\sin\psi_{z}\sin\phi_{x} \end{array}\\ \sin\psi_{z}\cos\theta_{y} & \begin{array}{c} \sin\psi_{z}\sin\theta_{y}\sin\phi_{x}\\ +\cos\psi_{z}\cos\phi_{x} \end{array} & \begin{array}{c} \sin\psi_{z}\sin\theta_{y}\cos\phi_{x}\\ -\cos\psi_{z}\sin\phi_{x} \end{array}\\ -\sin\theta_{y} & \cos\theta_{y}\sin\phi_{x} & \cos\theta_{y}\cos\phi_{x} \end{array}\right] \end{array}$$

Therefore, the Cartesian coordinates of the real points cloud $c_{re}$ obtained from the intersection between the fixed straight lines (d) and the rotated real plane $\left(P_{re}\right)$ are expressed as:

(5) $$\left(c_{re}\right):\left\{\begin{array}{c} \left[\begin{array}{c} x\\ y\\ z \end{array}\right]=\left[\begin{array}{c} t*\tan\alpha \\ t\\ t*\sqrt{\tan^{2}\alpha +1}*\tan\beta \end{array}\right]\;\mathrm{for}\;\left\{\begin{array}{c} 0^{\circ }<\alpha <90^{\circ }\;or\\ 270^{\circ }<\alpha <360^{\circ }\\ -90^{\circ }<\beta <90^{\circ }\\ t\geq 0 \end{array}\right.\\ \forall t=\frac{\left(ul^{\prime }-u^{\prime }l\right)hk^{\prime \prime }+\left(u^{\prime }k-uk^{\prime }\right)hl^{\prime \prime }+\left(lk^{\prime }-l^{\prime }k\right)hu^{\prime \prime }}{\left(k^{\prime }l^{\prime \prime }-l^{\prime }k^{\prime \prime }\right)\tan\alpha +\left(lk^{\prime \prime }-kl^{\prime \prime }\right)+\left(l^{\prime }k-lk^{\prime }\right)\sqrt{\tan\alpha^{2}+1}\tan\beta }\\ \left[\begin{array}{c} x\\ y\\ z \end{array}\right]=\left[\begin{array}{c} t*\tan\alpha \\ t\\ -t*\sqrt{\tan^{2}\alpha +1}*\tan\beta \end{array}\right]\mathrm{for}\;\left\{\begin{array}{c} 90^{\circ }<\alpha <270^{\circ }\\ -90^{\circ }<\beta <90^{\circ }\\ t\leq 0 \end{array}\right.\\ \forall t=\frac{\left(ul^{\prime }-u^{\prime }l\right)hk^{\prime \prime }+\left(u^{\prime }k-uk^{\prime }\right)hl^{\prime \prime }+\left(lk^{\prime }-l^{\prime }k\right)hu^{\prime \prime }}{\left(k^{\prime }l^{\prime \prime }-l^{\prime }k^{\prime \prime }\right)\tan\alpha +\left(lk^{\prime \prime }-kl^{\prime \prime }\right)-\left(l^{\prime }k-lk^{\prime }\right)\sqrt{\tan\alpha^{2}+1}\tan\beta } \end{array}\right.$$

where

(6) $$\left.\begin{array}{cc} k & =\cos\psi_{z}\cos\theta_{y}+\cos\psi_{z}\sin\theta_{y}\sin\phi_{x}-\sin\psi_{z}\cos\phi_{x}\\ l & =a\cos\psi_{z}\cos\theta_{y}+b\cos\psi_{z}\sin\theta_{y}\sin\phi_{x}-b\sin\psi_{z}\cos\phi_{x}\\ u & =\cos\psi_{z}\sin\theta_{y}\cos\phi_{x}+\sin\psi_{z}\sin\phi_{x}\\ k^{\prime } & =\sin\psi_{z}\cos\theta_{y}+\sin\psi_{z}\sin\theta_{y}\sin\phi_{x}+\cos\psi_{z}\cos\phi_{x}\\ l^{\prime } & =a\sin\psi_{z}\cos\theta_{y}+b\sin\psi_{z}\sin\theta_{y}\sin\phi_{x}+b\cos\psi_{z}\cos\phi_{x}\\ u^{\prime } & =\sin\psi_{z}\sin\theta_{y}\cos\phi_{x}+\cos\psi_{z}\sin\phi_{x}\\ k^{\prime \prime } & =-\sin\theta_{y}+\cos\theta_{y}\sin\phi_{x}\\ l^{\prime \prime } & =-a\sin\theta_{y}+b\cos\theta_{y}\sin\phi_{x}\\ u^{\prime \prime } & =\cos\theta_{y}\cos\phi_{x} \end{array}\right\}\forall a\;\mathrm{and}\;b\in R$$

### 2.3. Error Modeling in Polar and Cartesian Coordinates

In this section, the systematic and random errors $w_{\rho },w_{\alpha },w_{\beta }$ are taken in consideration as a source of error to represent the modeling of the additive white Gaussian noise for each polar coordinates ρ,α,β of the real points cloud $c_{re}$ in Equation (7), where $\rho_{w},\alpha_{w},\beta_{w}$ are the real measurements of the range ρ, azimuth α and elevation β, respectively, for each reflecting point.

(7) $$\left(c_{w}\right):\left[\begin{array}{c} \rho_{w}\\ \alpha_{w}\\ \beta_{w} \end{array}\right]=\left[\begin{array}{c} \rho +w_{\rho }\\ \alpha +w_{\alpha }\\ \beta +w_{\beta } \end{array}\right]$$

The 3D transformation from polar coordinates $\rho_{w},\alpha_{w},\beta_{w}$ to Cartesian coordinates $x_{w},y_{w},z_{w}$ of the ground noisy points cloud $c_{w}$ is given by:

(8) $$\left(c_{w}\right):\left[\begin{array}{c} x_{w}\\ y_{w}\\ z_{w} \end{array}\right]=\left[\begin{array}{c} \rho_{w}\cos\beta_{w}\sin\alpha_{w}\\ \rho_{w}\cos\beta_{w}\cos\alpha_{w}\\ \rho_{w}\sin\beta_{w} \end{array}\right]$$

Then, the standard deviation of the error can be derived from polar to Cartesian parameters in Equation (9), assuming that $\sigma_{x_{w}},\sigma_{y_{w}},\sigma_{z_{w}},\sigma_{\rho_{w}},\sigma_{\alpha_{w}},\sigma_{\beta_{w}}$ are, respectively, the standard deviations of the added noise on $x_{w},y_{w},z_{w},\rho_{w},\alpha_{w},\beta_{w}$. The terms of the standard deviations $\sigma_{\rho_{w}},\sigma_{\alpha_{w}},\sigma_{\beta_{w}}$ with a power higher than two can be neglected in this derivation to obtain this approximation:

(9) $$\left[\begin{array}{c} \sigma_{x_{w}}^{2}\\ \sigma_{y_{w}}^{2}\\ \sigma_{z_{w}}^{2} \end{array}\right]≃\left[\begin{array}{c} \sigma_{\rho_{w}}^{2}\cos^{2}\beta_{w}\sin^{2}\alpha_{w}+\rho_{w}^{2}\left(\sigma_{\beta_{w}}^{2}\sin^{2}\beta_{w}\sin^{2}\alpha_{w}+\sigma_{\alpha_{w}}^{2}\cos^{2}\beta_{w}\cos^{2}\alpha_{w}\right)\\ \sigma_{\rho_{w}}^{2}\cos^{2}\beta_{w}\cos^{2}\alpha_{w}+\rho_{w}^{2}\left(\sigma_{\beta_{w}}^{2}\sin^{2}\beta_{w}\cos^{2}\alpha_{w}+\sigma_{\alpha_{w}}^{2}\cos^{2}\beta_{w}\sin^{2}\alpha_{w}\right)\\ \sigma_{\rho_{w}}^{2}\sin^{2}\beta_{w}+\rho_{w}^{2}\sigma_{\beta_{w}}^{2}\cos^{2}\beta_{w} \end{array}\right]$$

Glennie et al. [20] showed in particular that the error of a scanning LiDAR sensor is mainly manifested over range. In this type of sensor, angles are not directly measured, but the error is mainly related to the reproducibility of the measurement for a given angle. The hypothesis of neglecting the scanning angle error is a very common assumption in the field of LiDAR detection: it is part of the manufacturers’ specifications and is commonly used in the literature. This is particularly related to the very small influence of the angle reproducibility errors on the range measurement of the object of interest.

In this study, we then focus on the range error $\sigma_{\rho_{w}}$ and neglect the azimuth and elevation errors $\sigma_{\alpha_{w}},\sigma_{\beta_{w}}$, respectively, in the simulation data as given by the constructor. The transformation in Equation (9) is then simplified as:

(10) $$\left[\begin{array}{c} \sigma_{x_{w}}\\ \sigma_{y_{w}}\\ \sigma_{z_{w}} \end{array}\right]=\left[\begin{array}{c} \sigma_{\rho_{w}}\cos\beta_{w}\sin\alpha_{w}\\ \sigma_{\rho_{w}}\cos\beta_{w}\cos\alpha_{w}\\ \sigma_{\rho_{w}}\sin\beta_{w} \end{array}\right]$$

## 3. LiDAR/Ground Extrinsic Calibration Method

In multi-sensor applications, data acquired from the different sensors must be fused in one common reference frame. In this application, the calibration of LiDAR frame scans is necessary to merge them in one world reference frame, in order to increase the points density coverage on the ground, which facilitates the road defect detection. Therefore, the extrinsic calibration aims to model the relationship between the LiDAR frame and the world reference frame.

**Figure 3:**
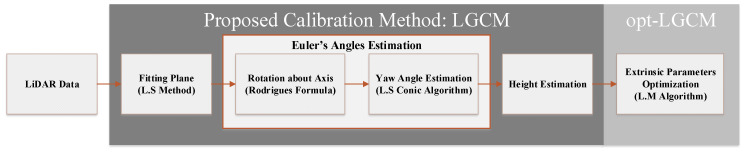
The proposed extrinsic calibration method block diagram.

We thus propose the LiDAR/Ground Calibration Method (LGCM) presented in Figure 3. The method includes the following procedures: fitting ground plane by Least Squares estimator, rotation about axis by Rodrigues formula, Least Squares Conic Algorithm, and height estimation. The proposed method is supplemented by Levenberg–Marquardt optimization algorithm as opt-LGCM. The main role of LGCM procedure is to estimate the extrinsic parameters: the Euler’s rotational angles $\psi_{z},\theta_{y},\phi_{x}$ and the height *h*. Then, the opt-LGCM is initialized by the estimated extrinsic parameters to optimize them. Finally, the distributed ground noisy points $c_{w}$ along the real plane $\left(P_{re}\right)$ are rotated along the horizontal plane $\left(P_{H}\right)$ by the optimized extrinsic parameters in the frame of fixed LiDAR.

The proposed method consists mainly in two steps. The first, totally unsupervised step, consists of estimating a first value of the steering angles. This first estimate is then used as a basis for the optimization step, which seeks the best orientation parameters. The developed method is therefore totally unsupervised and does not require a priori knowledge of the orientation of the sensor by a tilt unit for example.

### 3.1. Fitting Plane

The first step aims to fit an estimated plane $\left(P_{est}\right)$ with the rotated ground noisy points $c_{w}$. The Least Squares estimator is used to obtain the normal vector of the plane $\left(P_{est}\right)$.

The equation of the estimated plane $\left(P_{est}\right)$ in the LiDAR frame is expressed by:

(11) f(x,y)=z=Ax+By+D+w

where *A*, *B*, and *D* are the plane parameters and *w* is an additive white Gaussian noise with standard deviation $\sigma_{w}$.

Therefore, Equation (11) of the estimated plane $\left(P_{est}\right)$ can be written in linear form as:

(12) Z=HO+w

where

$Z={\left[\begin{array}{ccc} z(0) & \cdots & z(N-1) \end{array}\right]}^{T}$

$H=\left[\begin{array}{ccc} x(0) & y(0) & 1\\ \vdots & \vdots & \vdots \\ x(N-1) & y(N-1) & 1 \end{array}\right]$

$O={\left[\begin{array}{ccc} A & B & D \end{array}\right]}^{T}$

$w={\left[\begin{array}{ccc} w(0) & \cdots & w(N-1) \end{array}\right]}^{T}\;\;$ where *N* is the number of reflected points.

The solution of Least Squares estimator for this linear model is expressed as:

(13) $${\hat{O}}_{LS}={\left(H^{T}H\right)}^{-1}H^{T}Z$$

### 3.2. Rotation about Axis

Rodrigues formula is an efficient rotation transformation that computes the rotation matrix $R_{rod}$, which rotates a vector into another vector in 3D frame around a fixed axis vector $\vec{Axis}$ by rotational angle η [26]. Therefore, after having estimated the parameter vector of the oblique estimated plane $\left(P_{est}\right)$ according to Section 3.1, the next step is to compute the rotational matrix $R_{rod}$ from the normal vector ${\vec{n}}_{1}$ of the oblique estimated plane $\left(P_{est}\right)$ to the normal vector ${\vec{n}}_{2}$ of the horizontal plane $\left(P_{H}\right)$ that is parallel to $X_{{}_{L}}Y_{{}_{L}}$-plane with height −h (cf. Figure 1c). The objective of this step is to use Rodrigues formula in order to estimate the first two Euler’s angles pitch ${\hat{\phi }}_{x}$, roll ${\hat{\theta }}_{y}$, and the first partial yaw angle ${\hat{\Psi }}_{z1}$—due to the incomplete calibration in yaw rotation, which is solved by the next step—from Rodrigues Matrix $R_{rod}$.

Assuming that ${\vec{n}}_{1}$, ${\vec{n}}_{2}$, and $\vec{Axis}$ are expressed as:

${\vec{n}}_{1}\left(-\hat{A},-\hat{B},1\right)$

${\vec{n}}_{2}\left(0,0,1\right)$

$\vec{Axis}\left(m,n,p\right)=\frac{{\vec{n}}_{1}\times {\vec{n}}_{2}}{∥{\vec{n}}_{1}\times {\vec{n}}_{2}∥}$, the Rodrigues rotation formula $R_{rod}$ can be then written as:

(14) $$R_{rod}=I_{3}+\sin\eta K+\left(1-\cos\eta K^{2}\right)$$

where

$I_{3}=\left[\begin{array}{ccc} 1 & 0 & 0\\ 0 & 1 & 0\\ 0 & 0 & 1 \end{array}\right]$

$K=\left[\begin{array}{ccc} 0 & -p & n\\ p & 0 & -m\\ -n & m & 0 \end{array}\right]$

$\sin\eta =\frac{∥{\vec{n}}_{1}\times {\vec{n}}_{2}∥}{∥{\vec{n}}_{1}∥\cdot ∥{\vec{n}}_{2}∥}$

$\cos\eta =\frac{{\vec{n}}_{1}\cdot {\vec{n}}_{2}}{∥{\vec{n}}_{1}∥\cdot ∥{\vec{n}}_{2}∥}$

Now, by using Equation (15),

(15) $$R_{rod}=R_{x,y,z}\left({\hat{\Psi }}_{z1},{\hat{\theta }}_{y},{\hat{\phi }}_{x}\right)=R_{x}\left({\hat{\Psi }}_{z1}\right)R_{y}\left({\hat{\theta }}_{y}\right)R_{z}\left({\hat{\phi }}_{x}\right)$$

**Figure 4:**
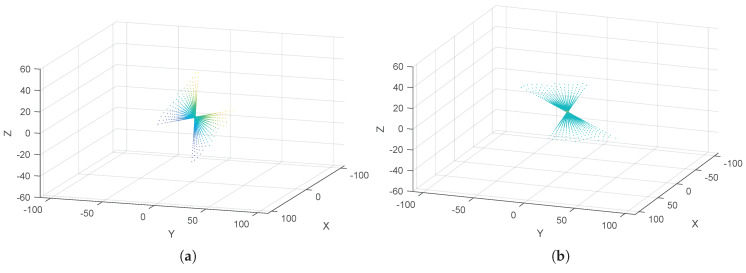
(**a**) Distributed ground noisy points $c_{w}$ about the real plane $\left(P_{re}\right)$; and (**b**) distributed points $c_{{}_{H1}}$ along the horizontal plane $\left(P_{H}\right)$.

Then, the Rodrigues matrix $R_{rod}$ provides the computation of ${\hat{\Psi }}_{z1}$, ${\hat{\theta }}_{y}$, and ${\hat{\phi }}_{x}$ as expressed in the equations below:

(16) $$\begin{array}{cc} {\hat{\Psi }}_{z1} & =\arctan\left({\left(R_{rod}\right)}_{21}/{\left(R_{rod}\right)}_{11}\right) \end{array}$$

(17) $$\begin{array}{cc} {\hat{\theta }}_{y} & =\arcsin(-{\left(R_{rod}\right)}_{31}) \end{array}$$

(18) $$\begin{array}{cc} {\hat{\phi }}_{x} & =\arctan\left({\left(R_{rod}\right)}_{32}/{\left(R_{rod}\right)}_{33}\right) \end{array}$$

where ij represents the matrix element index of ${\left(R_{rod}\right)}_{ij}$. As a graphical result, the ground noisy points $c_{w}$ are rotated by Rodrigues matrix $R_{rod}$ to the distributed points $c_{{}_{H1}}$ along the horizontal plane $\left(P_{H}\right)$ by Equation (19), as shown in Figure 4.

(19) $$c_{{}_{H1}}=R_{rod}c_{w}$$

### 3.3. Yaw Angle Estimation

**Figure 5:**
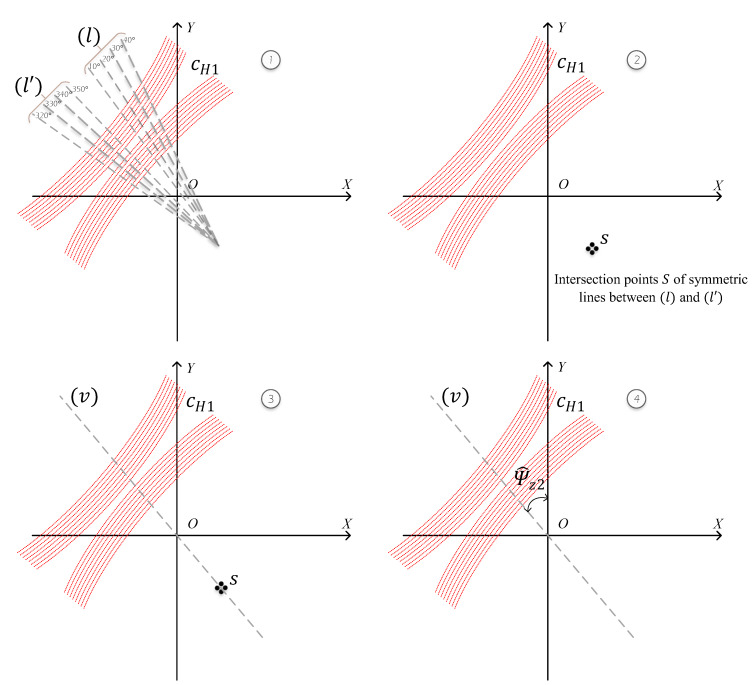
(**1**) Fitting the lines passing through the points of each $\zeta =10^{\circ }$ consecutive azimuth; (**2**) intersection points *S* of each symmetric lines between (l) and $\left(l^{\prime }\right)$; (**3**) fitting line (v) that passes through the points *S* and the origin *O*; and (4) angle ${\hat{\Psi }}_{z2}$ formed by line (v) and *y*-axis.

After rotating the noisy points $c_{w}$ to the horizontal points $c_{{}_{H1}}$, the second partial yaw angle ${\hat{\Psi }}_{z2}$ is estimated by the efficient Algorithm 1 that we propose in Figure 5 to rotate the points $c_{{}_{H1}}$ to $c_{{}_{H2}}$ about *z*-axis. This algorithm is called Least Squares Conic Algorithm (LSCA), which takes advantage of the center *s* characteristic of the geometrical impact patterns (hyperbolas, parabolas, and circles) formed by the points $c_{{}_{H1}}$, as shown in Figure 5. The aim of this part is to compute yaw angle ${\hat{\psi }}_{z}$ from the partial angles ${\hat{\Psi }}_{z1}$ and ${\hat{\Psi }}_{z2}$, as shown in Equation (20). 

**Algorithm 1:** Least Squares Conic Algorithm.   **Input:**
*x*,*y*,*z*,α,β of the distributed points $c_{{}_{H1}}$   **Output:**
${\hat{\Psi }}_{z2}$
1Fit the lines (l) and $\left(l^{\prime }\right)$ that pass through the points at each $\zeta =10^{\circ }$ consecutive azimuth by Least Squares estimator.The solution of Least Squares estimator for linear model:${\hat{O}}_{LS}={\left(H^{T}H\right)}^{-1}H^{T}Y$ where $Y=\left[\begin{array}{c} y(0)\\ \vdots \\ y(N-1) \end{array}\right]$, $H=\left[\begin{array}{cc} x(0) & 1\\ \vdots & \vdots \\ x(N-1) & 1 \end{array}\right]$, ${\hat{O}}_{LS}=\left[\begin{array}{c} \hat{m}\\ \hat{b} \end{array}\right]$2Compute the coordinates of the intersection points *s* of each two symmetric lines of (l) and $\left(l^{\prime }\right)$. Assume that:$\left(l\right):y={\hat{m}}_{1}x+{\hat{b}}_{1}$$\left(l^{\prime }\right):y={\hat{m}}_{2}x+{\hat{b}}_{2}$Therefore, the intersection points *s* of the straight lines (l) and $\left(l^{\prime }\right)$ are computed as follows:$x_{s}=\frac{{\hat{b}}_{2}-{\hat{b}}_{1}}{{\hat{m}}_{1}-{\hat{m}}_{2}}$,$y_{s}={\hat{m}}_{1}\frac{{\hat{b}}_{2}-{\hat{b}}_{1}}{{\hat{m}}_{1}-{\hat{m}}_{2}}+{\hat{b}}_{1}$3Fit a line (v) that passes through the intersection points *s* and the origin *O* by Least Squares estimator.The solution of Least Squares estimator: ${\hat{O}}_{LS}={\left(H^{T}H\right)}^{-1}H^{T}Y$ where $Y=\left[\begin{array}{c} y(0)\\ \vdots \\ y(N-1) \end{array}\right]$, $H=\left[\begin{array}{c} x(0)\\ \vdots \\ x(N-1) \end{array}\right]$, ${\hat{O}}_{LS}=\left[\begin{array}{c} \hat{m} \end{array}\right]$4Finally, compute the angle ${\hat{\Psi }}_{z2}$ formed by the fitting line (v) and *y*-axis:${\hat{\Psi }}_{z2}=\arctan\hat{m}-90^{\circ }\;\mathrm{if}\;\hat{m}>0$${\hat{\Psi }}_{z2}=\arctan\hat{m}+90^{\circ }\;\mathrm{if}\;\hat{m}<0$

Therefore, the third Euler’s angle of rotation (yaw angle) ${\hat{\psi }}_{z}$ is computed as follows:

(20) $${\hat{\psi }}_{z}={\hat{\Psi }}_{z1}-{\hat{\Psi }}_{z2}$$

**Figure 6:**
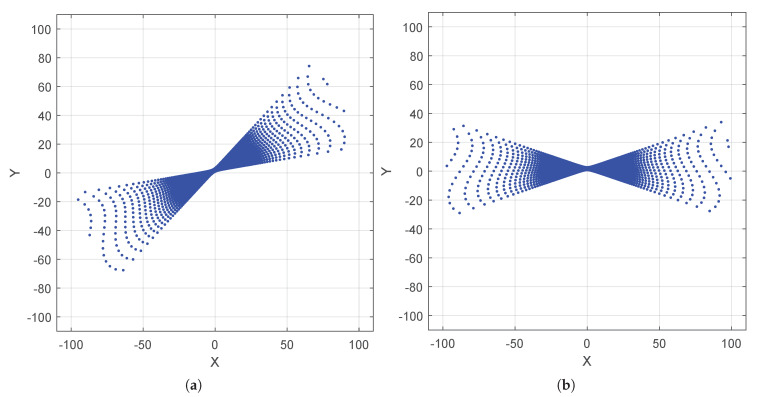
(**a**) Points $c_{{}_{H1}}$ before LSCA; and (**b**) points $c_{{}_{H2}}$ after LSCA rotated by ${\hat{\Psi }}_{z2}$ about *z*-axis.

Finally, the points $c_{{}_{H1}}$ are rotated to points $c_{{}_{H2}}$ by an angle $-{\hat{\Psi }}_{z2}$ around *z*-axis, as shown in Figure 6.

(21) $$c_{{}_{H2}}=R_{z}\left(-{\hat{\Psi }}_{z2}\right)c_{{}_{H1}}$$

### 3.4. Height Estimation

At the end of LGCM approach, a suitable way to estimate the height is to compute the altitude mean of the points $c_{{}_{H2}}$, due to the ground geometrical model used in this paper. Therefore, the estimated height is then expressed as:

(22) $$\hat{h}=\frac{1}{N}\sum_{i=0}^{N-1}z_{i}\;\mathrm{where}\;N\;\mathrm{is}\;\mathrm{the}\;\mathrm{number}\;\mathrm{of}\;\mathrm{calibrated}\;\mathrm{points}$$

### 3.5. Extrinsic Parameters Optimization

Levenberg–Marquardt algorithm is an optimization algorithm that combines gradient descent and Gauss–Newton methods [27]. In addition, it is a very efficient technique to find the minima and it performs well on most non-linear functions. Therefore, the role of opt-LGCM is to optimize the extrinsic parameters $\psi_{z},\theta_{y},\phi_{x},h$ by Levenberg–Marquardt algorithm, which is initialized by the estimated extrinsic parameters ${\hat{\psi }}_{z},{\hat{\theta }}_{y},{\hat{\phi }}_{x},\hat{h}$ to obtain the optimized extrinsic parameters ${\hat{\psi }}_{z}^{\prime \prime },{\hat{\theta }}_{y}^{\prime \prime },{\hat{\phi }}_{x}^{\prime \prime },{\hat{h}}_{opt}$, in order to minimize the mean square error mse that represents the square difference between the position of noisy points $c_{w}$ and the optimized position of points $c_{opt}$ in Equation (24). The optimized points $c_{opt}$ represent the intersection between all the LiDAR beams (d) and the optimized plane $\left(P_{opt}\right)$, which is the rotation of the horizontal plane $\left(P_{H}\right)$ by the new optimized Euler’s angles ${\hat{\psi }}_{z}^{\prime \prime },{\hat{\theta }}_{y}^{\prime \prime },{\hat{\phi }}_{x}^{\prime \prime }$ and the optimized height ${\hat{h}}_{opt}$ in Equation (25). In other words, the importance of the above procedure is to get the optimized height ${\hat{h}}_{opt}$ and the optimized Euler’s angles ${\hat{\psi }}_{z}^{\prime \prime },{\hat{\theta }}_{y}^{\prime \prime },{\hat{\phi }}_{x}^{\prime \prime }$ that rotate in the inverse ordering orientation the horizontal plane $\left(P_{H}\right)$, to fit the noisy points $c_{w}$ that are distributed along the oblique real plane $\left(P_{re}\right)$ with minimum mse on the position.

(23) $$\left({\hat{\psi }}_{z}^{\prime \prime },{\hat{\theta }}_{y}^{\prime \prime },{\hat{\phi }}_{x}^{\prime \prime },{\hat{h}}_{opt}\right)=\underset{\left(\psi_{z},\theta_{y},\phi_{x},h\right)}{\arg\min}mse$$

The non-linear function mse is expressed by:

(24) $$mse=\frac{1}{m}\sum_{i=1}^{m}\left(\begin{array}{c} {\left(x_{c_{opt}}-x_{c_{w}}\right)}^{2}+{\left(y_{c_{opt}}-y_{c_{w}}\right)}^{2}+{\left(z_{c_{opt}}-z_{c_{w}}\right)}^{2} \end{array}\right)$$

The optimized points $c_{opt}$ represent the intersection between the straight lines (d) and the rotated optimized plane $\left(P_{opt}\right)$, where the plane $\left(P_{opt}\right)$ is the rotation of the fixed ground horizontal plane $\left(P_{H}\right)$ of height ${\hat{h}}_{opt}$ by $-{\hat{\psi }}_{z}^{\prime \prime },-{\hat{\theta }}_{y}^{\prime \prime },-{\hat{\phi }}_{x}^{\prime \prime }$ based on $R_{x,y,z}$ rotation matrix, as shown in Equation (25):

(25) $$P_{opt}=R_{x,y,z}\left(-{\hat{\psi }}_{z}^{\prime \prime },-{\hat{\theta }}_{y}^{\prime \prime },-{\hat{\phi }}_{x}^{\prime \prime }\right)P_{H}$$

where the rotation matrix $R_{x,y,z}$ is the reverse of $R_{z,y,x}$.

(26) $$\begin{array}{c} R_{x,y,z}\left(-{\hat{\psi }}_{z}^{\prime \prime },-{\hat{\theta }}_{y}^{\prime \prime },-{\hat{\phi }}_{x}^{\prime \prime }\right)=R_{x}\left(-{\hat{\phi }}_{x}^{\prime \prime }\right)R_{y}\left(-{\hat{\theta }}_{y}^{\prime \prime }\right)R_{z}\left(-{\hat{\psi }}_{x}^{\prime \prime }\right)\\ =\left[\begin{array}{ccc} \cos{\hat{\psi }}_{x}^{\prime \prime }\cos{\hat{\theta }}_{y}^{\prime \prime } & \sin{\hat{\psi }}_{x}^{\prime \prime }\cos{\hat{\theta }}_{y}^{\prime \prime } & -\sin{\hat{\theta }}_{y}^{\prime \prime }\\ \begin{array}{c} \cos{\hat{\psi }}_{x}^{\prime \prime }\sin{\hat{\theta }}_{y}^{\prime \prime }\sin{\hat{\phi }}_{x}^{\prime \prime }\\ -\sin{\hat{\psi }}_{x}^{\prime \prime }\cos{\hat{\phi }}_{x}^{\prime \prime } \end{array} & \begin{array}{c} \sin{\hat{\psi }}_{x}^{\prime \prime }\sin{\hat{\theta }}_{y}^{\prime \prime }\sin{\hat{\phi }}_{x}^{\prime \prime }\\ +\cos{\hat{\psi }}_{x}^{\prime \prime }\cos{\hat{\phi }}_{x}^{\prime \prime } \end{array} & \cos{\hat{\theta }}_{y}^{\prime \prime }\sin{\hat{\phi }}_{x}^{\prime \prime }\\ \begin{array}{c} \cos{\hat{\psi }}_{x}^{\prime \prime }\sin{\hat{\theta }}_{y}^{\prime \prime }\cos{\hat{\phi }}_{x}^{\prime \prime }\\ +\sin{\hat{\psi }}_{x}^{\prime \prime }\sin{\hat{\phi }}_{x}^{\prime \prime } \end{array} & \begin{array}{c} \sin{\hat{\psi }}_{x}^{\prime \prime }\sin{\hat{\theta }}_{y}^{\prime \prime }\cos{\hat{\phi }}_{x}^{\prime \prime }\\ -\cos{\hat{\psi }}_{x}^{\prime \prime }\sin{\hat{\phi }}_{x}^{\prime \prime } \end{array} & \cos{\hat{\theta }}_{y}^{\prime \prime }\cos{\hat{\phi }}_{x}^{\prime \prime } \end{array}\right] \end{array}$$

Finally, the Cartesian coordinates of rotated optimized points cloud $c_{opt}$ in the reverse orientation sense are estimated by ${\hat{\psi }}_{z}^{\prime \prime },{\hat{\theta }}_{y}^{\prime \prime },{\hat{\phi }}_{x}^{\prime \prime },{\hat{h}}_{opt}$, as expressed below:

(27) $$\left(c_{opt}\right):\left\{\begin{array}{c} \left[\begin{array}{c} x\\ y\\ z \end{array}\right]=\left[\begin{array}{c} t*\tan\alpha \\ t\\ t*\sqrt{\tan^{2}\alpha +1}*\tan\beta \end{array}\right]\;\mathrm{for}\;\left\{\begin{array}{c} 0^{\circ }<\alpha <90^{\circ }\;or\\ 270^{\circ }<\alpha <360^{\circ }\\ -90^{\circ }<\beta <90^{\circ }\\ t\geq 0 \end{array}\right.\\ \forall t=\frac{\left(ul^{\prime }-u^{\prime }l\right){\hat{h}}_{opt}k^{\prime \prime }+\left(u^{\prime }k-uk^{\prime }\right){\hat{h}}_{opt}l^{\prime \prime }+\left(lk^{\prime }-l^{\prime }k\right){\hat{h}}_{opt}u^{\prime \prime }}{\left(k^{\prime }l^{\prime \prime }-l^{\prime }k^{\prime \prime }\right)\tan\alpha +\left(lk^{\prime \prime }-kl^{\prime \prime }\right)+\left(l^{\prime }k-lk^{\prime }\right)\sqrt{\tan\alpha^{2}+1}\tan\beta }\\ \left[\begin{array}{c} x\\ y\\ z \end{array}\right]=\left[\begin{array}{c} t*\tan\alpha \\ t\\ -t*\sqrt{\tan^{2}\alpha +1}*\tan\beta \end{array}\right]\mathrm{for}\;\left\{\begin{array}{c} 90^{\circ }<\alpha <270^{\circ }\\ -90^{\circ }<\beta <90^{\circ }\\ t\leq 0 \end{array}\right.\\ \forall t=\frac{\left(ul^{\prime }-u^{\prime }l\right){\hat{h}}_{opt}k^{\prime \prime }+\left(u^{\prime }k-uk^{\prime }\right){\hat{h}}_{opt}l^{\prime \prime }+\left(lk^{\prime }-l^{\prime }k\right){\hat{h}}_{opt}u^{\prime \prime }}{\left(k^{\prime }l^{\prime \prime }-l^{\prime }k^{\prime \prime }\right)\tan\alpha +\left(lk^{\prime \prime }-kl^{\prime \prime }\right)-\left(l^{\prime }k-lk^{\prime }\right)\sqrt{\tan\alpha^{2}+1}\tan\beta } \end{array}\right.$$

where

(28) $$\left.\begin{array}{cc} k & =\cos{\hat{\psi }}_{z}^{\prime \prime }\cos{\hat{\theta }}_{y}^{\prime \prime }+\sin{\hat{\psi }}_{z}^{\prime \prime }\cos{\hat{\theta }}_{y}^{\prime \prime }\\ l & =a\cos{\hat{\psi }}_{z}^{\prime \prime }\cos{\hat{\theta }}_{y}^{\prime \prime }+b\sin{\hat{\psi }}_{z}^{\prime \prime }\cos{\hat{\theta }}_{y}^{\prime \prime }\\ u & =-\sin{\hat{\theta }}_{y}^{\prime \prime }\\ k^{\prime } & =-\sin{\hat{\psi }}_{z}^{\prime \prime }\cos{\hat{\phi }}_{x}^{\prime \prime }+\cos{\hat{\psi }}_{z}^{\prime \prime }\sin{\hat{\theta }}_{y}^{\prime \prime }\sin{\hat{\phi }}_{x}^{\prime \prime }+\cos{\hat{\psi }}_{z}^{\prime \prime }\cos{\hat{\phi }}_{x}^{\prime \prime }+\sin{\hat{\psi }}_{z}^{\prime \prime }\sin{\hat{\theta }}_{y}^{\prime \prime }\sin{\hat{\phi }}_{x}^{\prime \prime }\\ l^{\prime } & =-a\sin{\hat{\psi }}_{z}^{\prime \prime }\cos{\hat{\phi }}_{x}^{\prime \prime }+a\cos{\hat{\psi }}_{z}^{\prime \prime }\sin{\hat{\theta }}_{y}^{\prime \prime }\sin{\hat{\phi }}_{x}^{\prime \prime }+b\cos{\hat{\psi }}_{z}^{\prime \prime }\cos{\hat{\phi }}_{x}^{\prime \prime }+b\sin{\hat{\psi }}_{z}^{\prime \prime }\sin{\hat{\theta }}_{y}^{\prime \prime }\sin{\hat{\phi }}_{x}^{\prime \prime }\\ u^{\prime } & =\cos{\hat{\theta }}_{y}^{\prime \prime }\sin{\hat{\phi }}_{x}^{\prime \prime }\\ k^{\prime \prime } & =\sin{\hat{\psi }}_{z}^{\prime \prime }\sin{\hat{\phi }}_{x}^{\prime \prime }+\cos{\hat{\psi }}_{z}^{\prime \prime }\sin{\hat{\theta }}_{y}^{\prime \prime }\cos{\hat{\phi }}_{x}^{\prime \prime }-\cos{\hat{\psi }}_{z}^{\prime \prime }\sin{\hat{\phi }}_{x}^{\prime \prime }+\sin{\hat{\psi }}_{z}^{\prime \prime }\sin{\hat{\theta }}_{y}^{\prime \prime }\cos{\hat{\phi }}_{x}^{\prime \prime }\\ l^{\prime \prime } & =a\sin{\hat{\psi }}_{z}^{\prime \prime }\sin{\hat{\phi }}_{x}^{\prime \prime }+a\cos{\hat{\psi }}_{z}^{\prime \prime }\sin{\hat{\theta }}_{y}^{\prime \prime }\cos{\hat{\phi }}_{x}^{\prime \prime }-b\cos{\hat{\psi }}_{z}^{\prime \prime }\sin{\hat{\phi }}_{x}^{\prime \prime }+b\sin{\hat{\psi }}_{z}^{\prime \prime }\sin{\hat{\theta }}_{y}^{\prime \prime }\cos{\hat{\phi }}_{x}^{\prime \prime }\\ u^{\prime \prime } & =\cos{\hat{\theta }}_{y}^{\prime \prime }\cos{\hat{\phi }}_{x}^{\prime \prime } \end{array}\right\}\forall a\;\mathrm{and}\;b\in R$$

## 4. Experimental Results

The proposed calibration method LGCM was applied on two types of data: simulation data were obtained by the modeling mentioned in Section 2 and real data acquisition from the Velodyne VLP-16 LiDAR. The most important features of Velodyne VLP-16 LiDAR for modeling are shown in Table 1.

The extrinsic calibration results are presented in terms of precision and robustness. According to our application, the precision shows the stability of the method with respect to the variation of pitch angle $\phi_{x}$ toward the ground, while the robustness shows the method strength with respect to the variation of range accuracy $\sigma_{\rho }$ of the measurements.

Therefore, the evaluation parameters of the results focus on the point cloud features of the real points $c_{re}$ on the real plane $\left(P_{re}\right)$, noisy points $c_{w}$ distributed along the real plane $\left(P_{re}\right)$, estimated points $c_{est}$ on the estimated plane $\left(P_{est}\right)$ obtained by LGCM, and the optimized points $c_{opt}$ on the optimized plane $\left(P_{opt}\right)$ obtained by opt-LGCM, as described below:

- The real height *h*, estimated height $\hat{h}$, and the optimized height ${\hat{h}}_{opt}$.
- The standard deviation $\sigma_{d_{w/i}}$ of the noisy points $c_{w}$ orthogonal Euclidean distance with respect to the real plane $\left(P_{re}\right)$, the estimated plane $\left(P_{est}\right)$ and the optimized plane $\left(P_{opt}\right)$.
  (29) $$\begin{array}{cc} \; & \sigma_{d_{w/i}}=\sqrt{\frac{1}{N}\sum {\left(d_{w/i}-\bar{d_{w/i}}\right)}^{2}} \end{array}$$
  (30) $$\begin{array}{cc} \; & d_{w/i}=\frac{|A_{i}x_{w}+B_{i}y_{w}+C_{i}z_{w}+D_{i}|}{\sqrt{A_{i}^{2}+B_{i}^{2}+C_{i}^{2}}} \end{array}$$
  where $x_{w},y_{w},z_{w}$ are the Cartesian coordinates of the noisy points $c_{w}$, $A_{i},B_{i},C_{i},D_{i}$ are the coefficient parameters of the planes, i={re,est,opt}, and *N* is the number of impact points.
- The standard deviation $\sigma_{\rho_{re}/\rho_{i}}$ of the real points $c_{re}$ range difference with respect to the noisy points $c_{w}$, the estimated points $c_{est}$, and the optimized points $c_{opt}$.
  (31) $$\sigma_{\rho_{re}/\rho_{i}}=\sqrt{\frac{1}{N}\sum {\left(\left(\rho_{re}-\rho_{i}\right)-\left(\bar{\rho_{re}-\rho_{i}}\right)\right)}^{2}}$$
  where i={w,est,opt} and *N* is the number of impact points.
- The standard deviation $\sigma_{\rho_{w}/\rho_{i}}$ of the noisy points $c_{w}$ range difference with respect to the real points $c_{re}$, the estimated points $c_{est}$, and the optimized points $c_{opt}$.
  (32) $$\sigma_{\rho_{w}/\rho_{i}}=\sqrt{\frac{1}{N}\sum {\left(\left(\rho_{w}-\rho_{i}\right)-\left(\bar{\rho_{w}-\rho_{i}}\right)\right)}^{2}}$$
  where i={re,est,opt} and *N* is the number of impact points.
- The gain in performance $PF_{i}$ that describes the range accuracy enhancement obtained from the Levenberg–Marquardt optimization algorithm which is defined as:
  (33) $$PF_{i}=\sigma_{\rho_{w}/\rho_{i}}-\sigma_{\rho_{w}/\rho_{re}}$$
  where $\sigma_{\rho_{w}/\rho_{re}}$ is the LiDAR range accuracy and i={est,opt}.

### 4.1. Simulation Data Results

Using the simulation data, the setups used to validate the proposed calibration method are separated in two categories:

- In term of precision, the real height h=2 m, roll angle $\theta_{y}=2^{\circ }$, yaw angle $\psi_{z}=2^{\circ }$, and LiDAR range accuracy $\sigma_{\rho_{w}/\rho_{re}}=0.03\;m$, with respect to the variation of pitch angle $\phi_{x}=\left[-70^{\circ },70^{\circ }\right]$.
- In term of robustness, the real height h=2 m, pitch angle $\phi_{x}=45^{\circ }$, roll angle $\theta_{y}=2^{\circ }$, and yaw angle $\psi_{z}=2^{\circ }$, with respect to the variation of $\sigma_{\rho_{w}/\rho_{re}}=\left[0,0.095\;m\right]$.

#### 4.1.1. Standard Deviation $\sigma_{d_{w/i}}$ in Terms of Precision and Robustness

**Figure 7:**
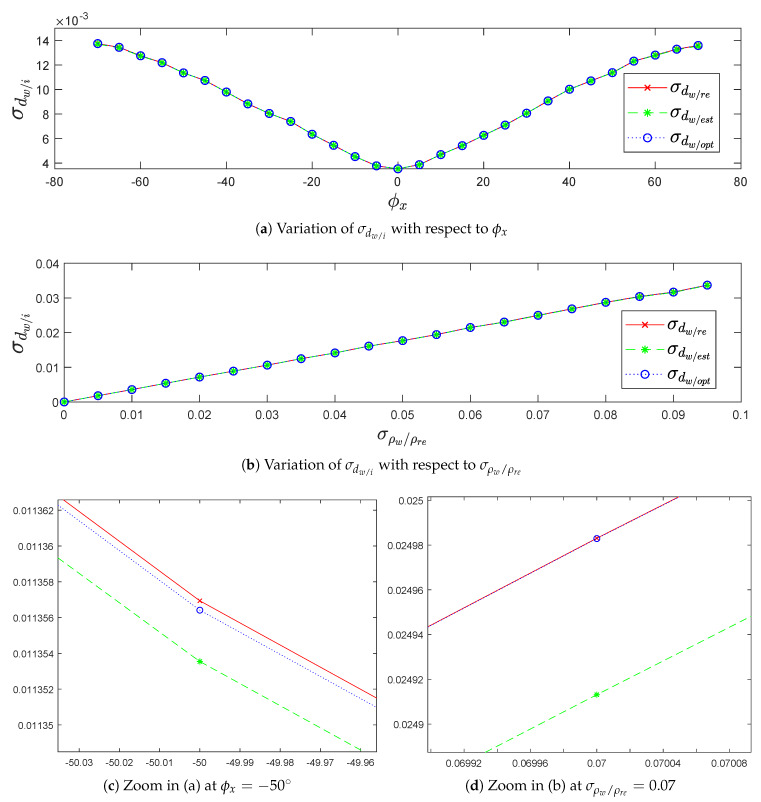
The variation of $\sigma_{d_{w/i}}$ in terms of precision and robustness.

After analyzing Figure 7a, the increasing of standard deviation $\sigma_{d_{w/i}}$ along the planes is due to the orientation effect of the LiDAR by the pitch angle $\phi_{x}$ on $\sigma_{d_{w/i}}$. Thus, as pitch angle $\phi_{x}$ tends to $90^{\circ }$, the standard deviation $\sigma_{d_{w/i}}$ tends to the LiDAR range accuracy $\sigma_{\rho_{w}/\rho_{re}}$. In Figure 7b, the increasing of the standard deviation $\sigma_{d_{w/i}}$ is due to increasing of LiDAR range accuracy $\sigma_{\rho_{w}/\rho_{re}}$. Moreover, Equation (34) describes the relation of $\sigma_{d_{w/i}}$ with $\phi_{x}$ and $\sigma_{\rho_{w}/\rho_{re}}$,which proves the increasing of $\sigma_{d_{w/i}}$.

(34) $$\sigma_{d_{w/i}}=\sin\left(\phi_{x}+\varphi_{k}\right)\sigma_{\rho_{w}/\rho_{re}}$$

where $\varphi_{k}$ is the elevation angle of each VLP-16 LiDAR laser, and k={1,2,…,16} represents the laser index.

In Figure 7c,d, we can see that the standard deviation $\sigma_{d_{w/opt}}$ is closer to the the standard deviation $\sigma_{d_{w/re}}$ than the standard deviation $\sigma_{d_{w/est}}$. This shows that the optimized plane $\left(P_{opt}\right)$ is better fit to the real plane $\left(P_{re}\right)$ than the estimated plane $\left(P_{est}\right)$.

#### 4.1.2. Standard Deviation $\sigma_{\rho_{re}/\rho_{i}}$ and $\sigma_{\rho_{w}/\rho_{i}}$ in Terms of Precision and Robustness

**Figure 8:**
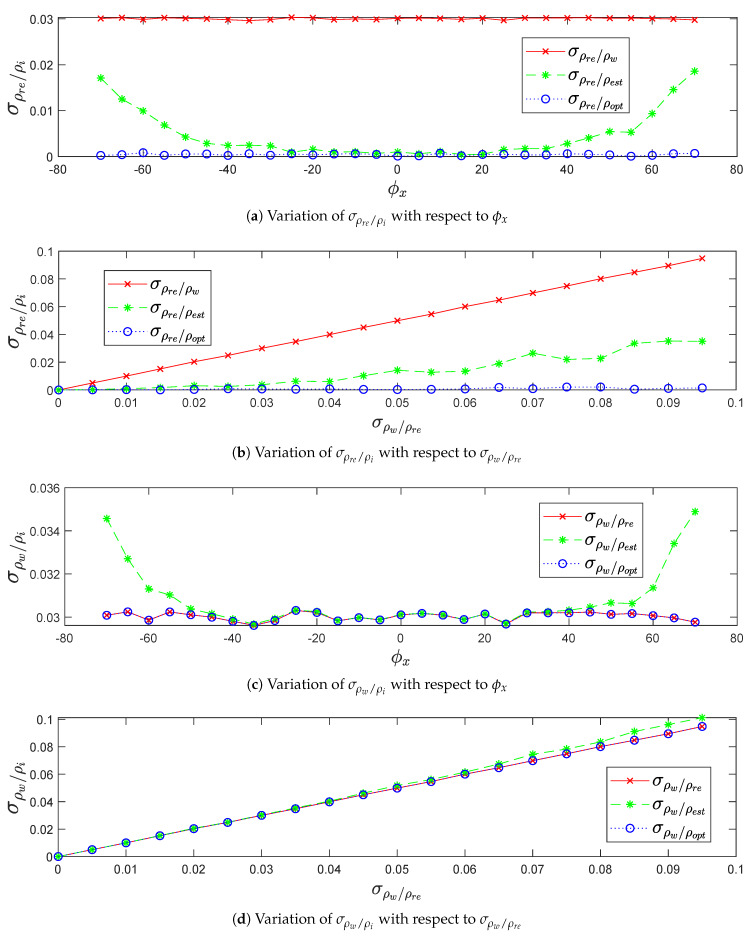
The variation of $\sigma_{\rho_{re}/\rho_{i}}$ and $\sigma_{\rho_{w}/\rho_{i}}$ in terms of precision and robustness.

In terms of precision and robustness, Figure 8 shows the increasing behavior of the range standard deviations $\sigma_{\rho_{re}/\rho_{est}}$ and $\sigma_{\rho_{w}/\rho_{est}}$ after the LGCM calibration, due to:

- The increase of pitch angle $\phi_{x}$ on positive and negative sides decreases the sparsity of impact points on the ground. This leads to decrease the precision of plane fitting estimation, as shown in Figure 8a,c.
- The increase of LiDAR range accuracy $\sigma_{\rho_{w}/\rho_{re}}$ decreases the precision of plane fitting estimation, as shown in Figure 8b,d.

The standard deviation $\sigma_{\rho_{re}/\rho_{opt}}$ is lower than the standard deviation $\sigma_{\rho_{re}/\rho_{est}}$, as shown in Figure 8a,b, which indicate how the optimized points $c_{opt}$ are closer to the real points $c_{re}$ than the estimated points $c_{est}$. On the other hand, the standard deviation $\sigma_{\rho_{w}/\rho_{opt}}$ is closer to LiDAR range accuracy $\sigma_{\rho_{w}/\rho_{re}}$ than the standard deviation $\sigma_{\rho_{w}/\rho_{est}}$, as shown in Figure 8c,d, which indicate the equality of the noisy points $c_{w}$ range distribution along the real plane $\left(P_{re}\right)$ and the optimized plane $\left(P_{opt}\right)$.

The negligible standard deviation $\sigma_{\rho_{re}/\rho_{opt}}$ in Figure 8a,b and the coincident standard deviations $\sigma_{\rho_{w}/\rho_{opt}}$ and $\sigma_{\rho_{w}/\rho_{re}}$ in Figure 8c,d prove the similarity of the real plane $\left(P_{re}\right)$ and the optimized plane $\left(P_{opt}\right)$ compare to the estimated plane $\left(P_{est}\right)$.

#### 4.1.3. Height Recovering in Terms of Precision and Robustness

**Figure 9:**
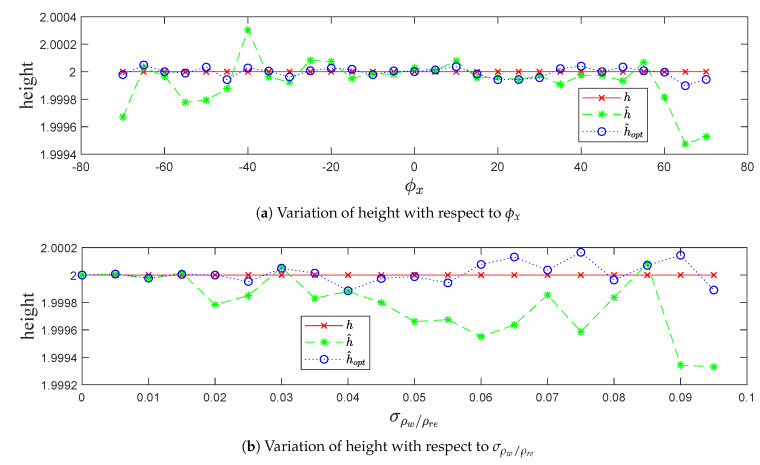
Height recovering in terms of precision and robustness.

In terms of precision and robustness, Figure 9 highlights the recovering of the height parameter and how the optimized height ${\hat{h}}_{opt}$ is closer to the real height *h* than the estimated height $\hat{h}$, which presents the height optimization importance and the strength of the Levenberg–Marquardt optimization algorithm.

#### 4.1.4. Performance Gain $PF_{i}$ in Terms of Precision and Robustness

**Figure 10:**
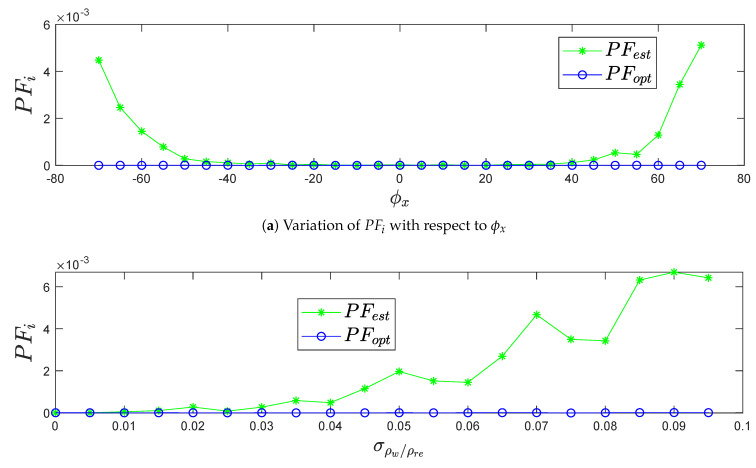
The variation of $PF_{i}$ in terms of precision and robustness

Figure 10 shows the gain in performance of the optimized plane points $c_{opt}$ against the estimated plane points $c_{est}$ distributed by the noisy points $c_{w}$ compared to the LiDAR range accuracy $\sigma_{\rho_{w}/\rho_{re}}$ as expressed in Equation (33), with respect to the variation of pitch angle $\phi_{x}$ and LiDAR range accuracy $\sigma_{\rho_{w}/\rho_{re}}$. Moreover, the negligibility of the method performance $PF_{opt}$ appears after the optimization, which means that the standard deviation $\sigma_{\rho_{w}/\rho_{opt}}$ after optimization is closer to the standard deviation $\sigma_{\rho_{w}/\rho_{est}}$ before optimization with respect to the LiDAR range accuracy $\sigma_{\rho_{w}/\rho_{re}}$. In addition, it presents the recovering of noisy points $c_{w}$ range distribution along the real plane $\left(P_{re}\right)$ after the optimization algorithm, taking advantage of maintaining the standard deviation $\sigma_{\rho_{w}/\rho_{opt}}$ value as negligible. The gain feature $PF_{i}$ proves again the better fit between the optimized plane $\left(P_{opt}\right)$ and the real plane $\left(P_{re}\right)$ rather than the estimated plane $\left(P_{est}\right)$.

### 4.2. Real Data Results

**Figure 11:**
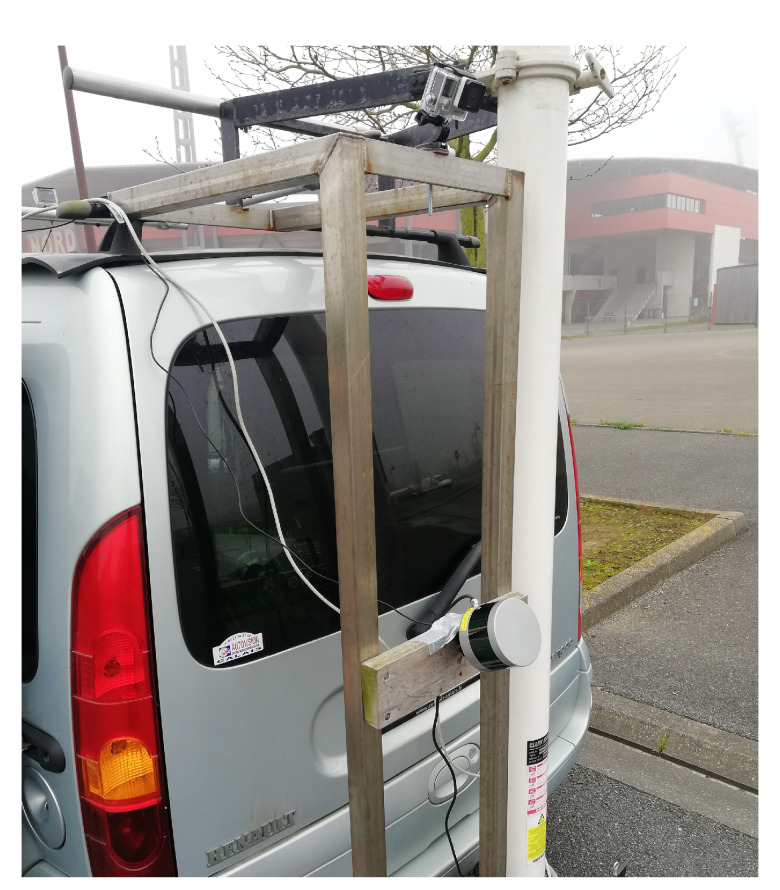
VLP-16 LiDAR mounted on a vehicle toward the ground.

The 3D point cloud acquisitions were obtained using a multi-lasers rangefinder VLP-16 LiDAR mounted on a vehicle. To obtain a telemetric information about the ground surface and to achieve the application goal, the VLP-16 LiDAR was rotated toward the ground direction with a pitch angle $\phi_{x}≃70^{\circ }$, and it was at a height of h≃1.05 m above the ground surface. The real setup is shown in Figure 11.

The proposed method was applied to two different acquisitions:

- Acquisition 1: The vehicle was at rest on the road.
- Acquisition 2: The vehicle was moving at a slow speed on the road.

#### Standard Deviation $\sigma_{\rho_{w}/\rho_{i}}$ per LiDAR Frames

**Figure 12:**
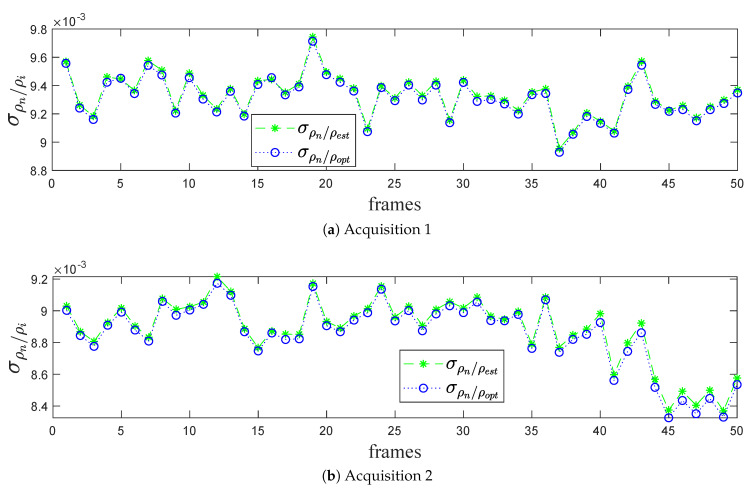
The variation of $\sigma_{\rho_{w}/\rho_{i}}$ with respect to LiDAR frame.

In the absence of real plane $\left(P_{re}\right)$ when using real data, the results focus on the range distribution of the noisy points $c_{w}$ along the estimated plane $\left(P_{est}\right)$ and the optimized plane $\left(P_{opt}\right)$. It is clear that the standard deviations $\sigma_{\rho_{w}/\rho_{opt}}$ curve is lower than the $\sigma_{\rho_{w}/\rho_{est}}$ in the two acquisitions, as shown in Figure 12. The optimization algorithm is thus proved to be more efficient for real data as well in decreasing the range distribution of the noisy points $c_{w}$ along the fitting planes.

### 4.3. Results Discussion

**Figure 13:**
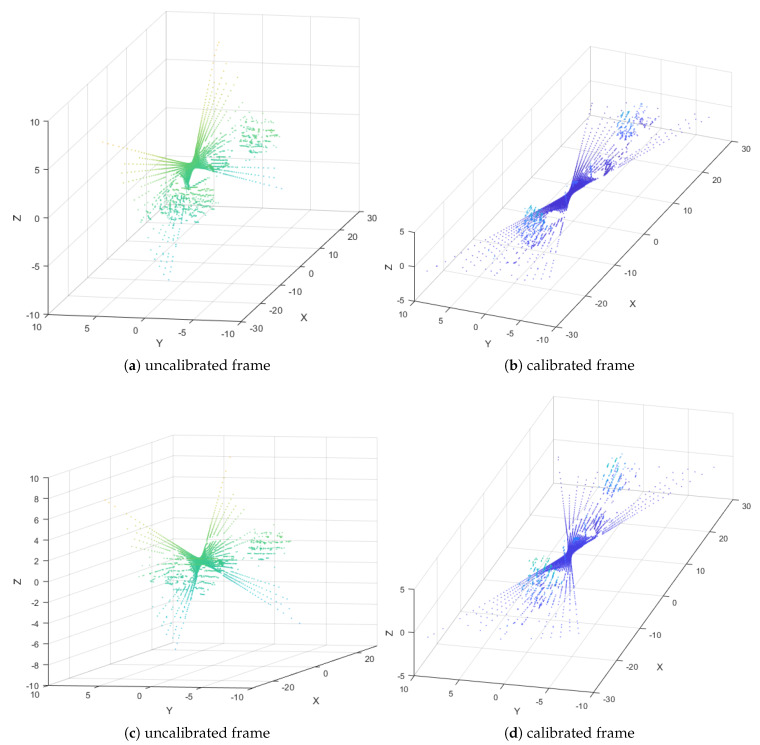
Uncalibrated and calibrated LiDAR frames from Acquisitions 1 and 2.

In general, the results prove the efficiency of the optimization algorithm, which is represented by the optimized plane $\left(P_{opt}\right)$, versus the estimated plane $\left(P_{est}\right)$, compared with the real plane $\left(P_{re}\right)$, in terms of precision and robustness. On the other hand, the convergence of the optimization algorithm is granted automatically by the suitable initialization parameters, namely the estimated Euler’s angles ${\hat{\psi }}_{z},{\hat{\theta }}_{y},{\hat{\phi }}_{x}$ and the estimated height $\hat{h}$, which are computed in Stage 1 (LGCM) to obtain the estimated plane $\left(P_{est}\right)$, and then optimized by Levenberg–Marquardt optimization algorithm (opt-LGCM) in Stage 2 to get the optimized Euler’s angles ${\hat{\psi }}_{z}^{\prime \prime },{\hat{\theta }}_{y}^{\prime \prime },{\hat{\phi }}_{x}^{\prime \prime }$ and the optimized height ${\hat{h}}_{opt}$ to obtain the optimized plane $\left(P_{opt}\right)$. Finally, the results show the strength and the method performance in terms of precision and robustness against the variation of pitch angle $\phi_{x}$ and LiDAR range accuracy $\sigma_{\rho_{w}/\rho_{re}}$, respectively, in order to achieve the application’s aim, as shown in Figure 13.

## 5. Conclusions

A new extrinsic LiDAR/Ground calibration method for 3D LiDARs is presented in this paper. The solution relies on plane-based modeling of the ground, which allows the estimation of the LiDAR’s orientation and altitude using Rodrigues formula, Least Squares Conic Algorithm for yaw angle estimation and height estimation. The proposed method (LGCM) is extended to an optimized derivation (opt-LGCM) using the Levenberg–Marquardt algorithm and is shown to be a suitable solution to LiDAR/Ground calibration problem. It is implemented on synthetic and real LiDAR telemetric data. The results show the performance in terms of precision and robustness against the variation of LiDAR’s orientation and range accuracy, respectively, proving the stability and the accuracy of the proposed calibration method.

## Figures and Tables

**Table 1:** VLP-16 features.

Features	VLP-16
Laser beams	16
Horizontal FOV	$360^{\circ }$
Vertical FOV	$-15^{\circ }\to +15^{\circ }$
Azimuth angular resolution	$0.1^{\circ }\;–\;0.2^{\circ }\;–\;0.4^{\circ }$
Elevation angular resolution	$2^{\circ }$
Maximum range accuracy $\sigma_{\rho }$	3 cm
